# The effects of injection of bovine vaccine into a human digit: a case report

**DOI:** 10.1186/1476-069X-4-21

**Published:** 2005-10-11

**Authors:** Jennifer K O'Neill, Simon W Richards, David M Ricketts, Marc H Patterson

**Affiliations:** 1 Orthopaedic Department, Princess Royal Hospital, Lewes Road, Haywards Heath, West Sussex RH16 4EX, UK

## Abstract

**Background:**

The incidence of needlestick injuries in farmers and veterinary surgeons is significant and the consequences of such an injection can be serious.

**Case presentation:**

We report accidental injection of bovine vaccine into the base of the little finger. This resulted in increased pressure in the flexor sheath causing signs and symptoms of ischemia. Amputation of the digit was required despite repeated surgical debridement and decompression.

**Conclusion:**

There have been previous reports of injection of oil-based vaccines into the human hand resulting in granulomatous inflammation or sterile abscess and causing morbidity and tissue loss.

Self-injection with veterinary vaccines is an occupational hazard for farmers and veterinary surgeons. Injection of vaccine into a closed compartment such as the human finger can have serious sequelae including loss of the injected digit. These injuries are not to be underestimated. Early debridement and irrigation of the injected area with decompression is likely to give the best outcome. Frequent review is necessary after the first procedure because repeat operations may be required.

## Background

Calf diarrhoea is a contagious and often fatal disease of calves. Rotavirus, Coronavirus and E. Coli are three of the most important causal agents.

The combined bovine Rotovirus, Coronavirus and E.coli F5 (K99) vaccine is an inactivated vaccine for calf diarrhoea. It is presented in an oil based emulsion adjuvant to boost the efficacy and duration of the vaccine. Vaccination is carried out by a 2 ml intramuscular injection. A single injection is given to pregnant cows and heifers 3–12 weeks before calving is expected [1].

Injection of this vaccine into a closed compartment such as the human finger can have serious sequelae. The oil-based nature of the vaccine means that it has the potential to cause increased compartmental pressure [2]. The entire finger (especially the finger pulp and tendon sheaths) is at risk. The information leaflet supplied with the vaccine [1] and a WHO report [2] suggests the user is strongly advised to obtain prompt surgical attention after accidental self-injection; early debridement and irrigation of the injected area may be necessary.

## Case Presentation

A 20-year-old herdsman accidentally injected his right little finger with the combined bovine vaccine. Approximately 1 ml of vaccine was injected under hand injecting pressure. One hour after injury he attended the Accident and Emergency department and was treated with oral Augmentin and a sling to elevate the hand. Four hours after the injury the patient returned as the finger was increasingly swollen and painful. On examination his finger was swollen, tense and tender. The capillary refill was less than two seconds. Sensation was decreased in the finger when compared to the little finger on the contralateral hand. A needle puncture site was noted at the base of the little finger on the palmar side over the proximal interphalangeal joint skin crease. Radiographs of the hand were normal. The white cell count was 16.2 × 10^9^/l (normal range 4–11 × 10^9^/l) with the differential showing both raised neutrophils at a count of 12.2 × 10^9^/l (normal range 2–7.5 × 10^9^/l) and monocytes at a count of 1 × 10^9^/l (normal range 0.2–0.8 × 10^9^/l).

Surgical decompression was undertaken through a Brunner incision. The flexor sheath was windowed in the A3 pulley. Lipid based fluid was released under high pressure. A further window was made at the A5 level and the flexor sheath was thoroughly irrigated. The skin incisions were left open. The hand was dressed and placed in a back-slab plaster in the 'position of safety'. The hand was elevated and neurovascular observations were performed regularly on the ward. The patient received intravenous Cefuroxine antibiotic. Forty-eight hours later secondary closure of the wound was performed in theatre and the patient was discharged four days after admission with a supply of oral Cephalosporin antibiotics.

Two days later the patient represented with a wound infection. The wound was discharging pus and he had tracking lymphangitis to the axilla. He was apyrexial and his white cell count was within the normal range but his CRP was raised to 11 (normal Value <9). In addition, a non-itchy rash with circular macules had developed over his chest and neck. The rash was thought to be a reaction to the Cephalosporin antibiotic. The arm was elevated in a Bradford sling and the antibiotics were changed to intravenous Flucloxacillin and Benzlypenicillin. The rash appeared to improve. Five days later the lymphangitis had receded and the wound was clean. The patient was discharged on oral Flucloxacillin and Penicillin antibiotics. Microbiology cultures of the fluid removed in theatre, the pus from the wound and blood cultures were all negative for organisms.

The patient was readmitted three days later with recurrence of lymphangitis. Although he was still apyrexial and his white cell count remained within the normal range, his CRP had now risen to 126. The wound was again debrided in theatre and oily white fluid was found in the flexor sheath. The central part of the wound was left open and packed with saline soaked ribbon gauze. Cultures still showed no growth of any organism.

The pain and swelling continued and a week later he was referred to plastic surgery. At operation, there was necrotic tissue around the middle and proximal phalanges of the little finger, extending into the tendons and around the head of the fifth metacarpal. The radial neurovascular bundle could not be located. Amputation of the finger at the metocarpophalangeal joint was performed. No histopathological examination was performed on the specimen but microbiological examination of the specimen did not reveal any pathogenic organisms.

Two months after the amputation the patient was seen at a follow up appointment complaining of some pain in the fifth metacarpal of the right hand a feeling of stiffness of the joints of the right hand. The story was complicated by a recent injury to the hand as he crushed it between a chair and a heavy table three days prior to the appointment. He also had a recurrence of the rash on his chest. A dermatologist opinion diagnosed the rash as pityriasis versicolor due to a malassezia furfur infection on the skin and this was proven by skin biopsies. It was considered by the dermatologist that an atypical mycobacteria infection of the hand (possibly from soil encountered on the farm) might have been missed on routine histopathology and culture. To treat both this possibility and the pityriasis, Tetralysal tablets were started. This treatment was continued for two months and good progress was made with resolution of symptoms.

## Discussion

Self injection with veterinary vaccines is an occupational hazard for farmers and veterinary surgeons. A survey of veterinary surgeons reported the rate of needlestick injuries at 5.5/100 vets per year or 1 in 1000 cases of vaccine given [3]. The hand was the site of injury in 17% of cases.

In a closed compartment such as the flexor sheath of a digit injected oil adjuvant based vaccine may have disastrous consequences. In a similar way, high pressure injections of paint [4,5], grease or diesel oil [5,6] or dry cleaning solvents [7] may lead to severe and irreversible loss of function or amputation [8] due to increased pressure within the closed space [8] or subsequent infection [9]. The high pressure injecting devices used in industry [5] and those that deliver a fixed volume per injection [10] are thought to be particularly dangerous but in this case the patient was using a simple 2 ml syringe and 23 guage needle.

Oil based veterinary vaccines have previously been reported to cause a prolonged chronic granulomatous reaction with sterile abscess formation [11] that may result in significant morbidity requiring multiple operations for debridement [11].

Amputation of the dominant thumb at the metacarpophalangeal joint has been reported following an injection of a pig parvovirus vaccine [10] and another patient, following injection of fowl pest vaccine, lost the terminal phalanx of a finger [12]. Other long-term sequelae include neuralgic pain and cold intolerance at the site of the injection. These vaccinations are also delivered at hand pressure and the lack of a high pressure injecting system does not negate serious sequelae of these injections. The outcome appears to be related to the volume injected. These injuries are often under-estimated [9] and delayed diagnosis and debridement may occur. The use of systemic steroids to decrease the swelling is controversial (as it may increase the risk of infection) but should be considered if oedema is significant [7].

## Conclusion

Our case involves injection of a small amount of bovine vaccine into the base of the little finger. The swelling and reaction caused by oil trapped within the flexor sheath was likely to be causing compression of the neurovascular supply to the finger. Despite decompression, further ischemia and necrosis of the digit occurred. In addition, there may have been an infection, even though a pathologic agent was never cultured by microbiological tests. Amputation was required. There are no clear guidelines available to guide practicing clinicians in deciding whether to explore and debride such a wound. It has been suggested that injections of a small amount of vaccine can be treated conservatively [4]. However, this case illustrates the seriousness of such an injury despite early surgical treatment. These injuries are not to be underestimated. Early debridement and irrigation of the injected area with decompression is likely to give the best outcome [13]. Frequent review is necessary after the first procedure because repeat operations may be required.

## Competing interests

The author(s) declare that they have no competing interests.

## Authors' contributions

Jennifer O'Neill carried out the literature searches and is the main author, Simon Richards was involved with writing and editing, David Ricketts was involved with editing and revising the paper and Marc Patterson was involved with editing and revising the paper.
